# A legal battle between Ecuadorian citizens and corporate power

**DOI:** 10.7189/jogh.15.03017

**Published:** 2025-05-12

**Authors:** Ivan Sisa, María Belen Mena

**Affiliations:** 1 Escuela de Medicina, Universidad San Francisco de Quito USFQ, Quito, Ecuador; 2 Facultad de Ciencias Médicas, Universidad Central del Ecuador, Quito, Ecuador

## Abstract

For decades, transnational corporations/companies have been using various strategies (*e.g.* lobbying for the reduction of taxation) to position their products and influence dietary habits, particularly in low- and middle-income countries. According to the World Health Organization (WHO), ‘health taxes’ are one of the most effective public health measures for reducing the negative effects of unhealthy products, which have been shown to add to the burden adiposity-related cardiovascular diseases, cancers, and diabetes mellitus. Yet despite the abundant evidence on these impacts and the innovative tax policies implemented by neighbouring countries, on 10 January 2023, Ecuador issued *Decreto 645* – an unprecedented presidential decree which reduced excise taxes on products harmful to health and the environment, including cigarettes and e-cigarettes, alcoholic beverages, sugary industrial drinks, plastic covers, firearms, and ammunition. The rationale for this government decree was to strengthen citizen security, fight against smuggling and informality, and mitigate the impact of inflation on the economy. In this viewpoint, we share the experience of how members of the civil society built a group called *Colectivo Todos Por la Vida *(translated as ‘Collective All for Life’) and led a legal fight to defend the health and well-being of the Ecuadorian population against local corporate power. The lessons learned could be useful to other countries in similar scenarios.

## HEALTH TAXES AND THEIR IMPACT

**Figure 1:**
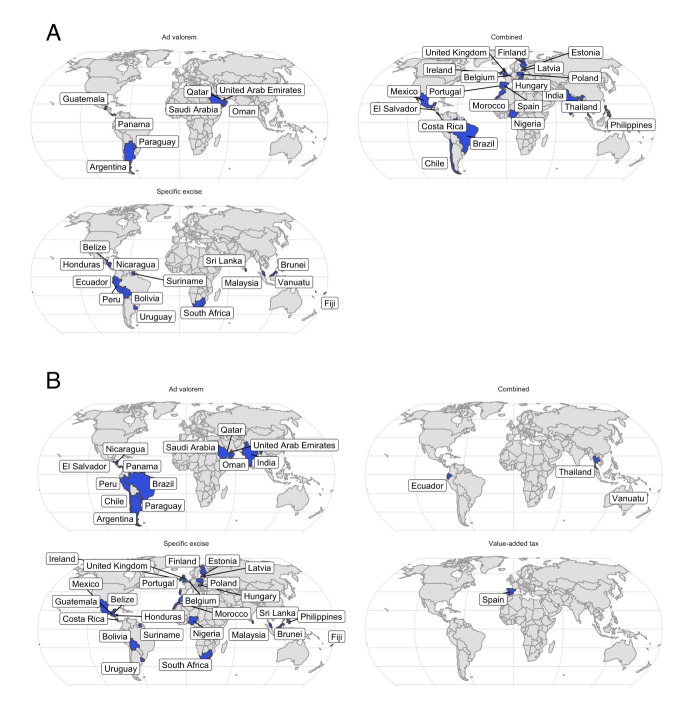
Health taxes implementation across countries. Panel A. Tobacco taxes. Panel B. SSB taxes. *Ad valorem* is applied as a percentage of the value of the product [7]. Specific excise is applied as a specific amount per unit volume or may be based on beverage characteristics rather than as a function of the value of the product [7]. Value-added tax is levied on the price of a product or service at each stage of production, distribution and sale to the final consumer [14]. Combined may be a mix of excise and import tariffs or excise and *ad valorem*. Graphic created using RStudio, version 2024.04.2 (Posit, Boston, Massachusetts, USA) and information from the following sources [6–9,15].

According to the World Health Organization (WHO), increasing the prices of harmful products by increasing taxes (*i.e.* implementing so-called ‘health taxes’) has proven to be one of the most effective public health measures for mitigating their impact on population health [1]. This strategy discourages children, adolescents, and low-resource populations from consuming these harmful products, which then reflects in fewer diseases, disabilities, and deaths [1]. On the manufacturing side, taxes can incentivise companies to reformulate their products and offer consumers healthier food choices. This simultaneously addresses issues of market failures and health inequalities, as less affluent socioeconomic groups tend to be more reactive to price increases, meaning they would likely reduce their consumption of unhealthy dietary products [2]. However, economic and political forces shape diet, nutrition, and health of populations worldwide, particularly those in low- and middle-income countries (LMICs) [3]. For at least the past 50 years, transnational corporations/companies have been using various strategies to position their products, including marketing of unhealthy products, influencing government policymaking, funding public health campaigns, and lobbying for tax reductions [4]. Transnational corporations deprive countries of at least USD 245 billion in taxes each year through tax havens [5]. Simultaneously, taxation – a well-known countermeasure for reducing the negative effects of unhealthy products – has been implemented by several countries worldwide [4]. For example, taxes on tobacco and sugar-sweetened beverages (SSBs) have been implemented in various modalities (*e.g. ad valorem*, specific excise, value-added tax, and combined modalities) over the past three decades and in more than 50 countries to date [6–9]. Historically, governments have used tobacco taxes to raise revenue and finance wars since 1790 [10]. However, tax initiatives to prevent tobacco consumption for reasons of protecting population health date back to 1988, when citizens of California, USA, succeeded in placing a powerful tobacco tax called ‘Proposition 99’. This initiative increased the tax on cigarettes by 25 cents per pack, or more precisely, from 10 to 35 cents in 1989, from 35 to 37 cents in 1994, and from 37 to 87 cents in 1999 [11,12]. In the first few years following its implementation, smoking prevalence in California declined from 26.8% in 1987 to 22.2% in 1990, while cigarette sales declined by 9.4% [11]. Since then, several other countries have copied this initiative by implementing tobacco taxation, despite strong opposition from the tobacco industry [13] **(****Figure 1**, Panel A). The literature shows that the implementation of tobacco taxation is the most cost-effective strategy for modifying consumer behaviour and health outcomes, and for generating revenues for health systems across all settings [13,16]. For example, raising tobacco taxes to achieve an average increase in prices by 10% is estimated to reduce smoking prevalence by 4% in high-income countries and by 5% in LMICs. If all countries increased taxes on cigarette packs by 50%, they would manage to avert 11 million deaths (*e.g.* from lung cancer and cardiovascular diseases), while governments would generate an additional USD 101 billion in revenue that could be used to strengthen local health care systems to prevent/control noncommunicable diseases [13,16].

Similarly, various types of taxes on SSBs have been implemented worldwide to improve population nutrition and health **(****Figure 1**, Panel B**)**. Andreyeva and colleagues conducted a meta-analysis of outcomes following taxation of SSBs and found that the demand for SSBs was highly sensitive to tax-induced price increases, with a mean reduction in SSBs sales of 15% (95% confidence interval = −20, −9; *P* < 0.001) [17] Additionally, a recent comparative risk assessment model estimated that 2.2 million new cases of type 2 diabetes (T2D) and 1.2 million new cases of cardiovascular diseases (CVDs) were attributable to SSBs worldwide in 2020, with Latin America and the Caribbean having the highest absolute number of cases per million adults (*i.e.* persons aged ≥20 years) [18]. The T2D and CVDs related to SSB consumption are caused either directly through weight gain or through a pathway mediated by it. If prevention and treatment measures do not improve by 2035, the global economic impact of overweight/obesity will reach $4.32 trillion annually. If current trends continue, nearly two billion population – most of whom will reside in LMICs – will be obese by 2035 [19]. Due to these negative effects on the health and well-being of the population stemming from the consumption of harmful products (including tobacco and foods high in fat, salt, and sugar), some countries have been seeking more aggressive and comprehensive efforts to influence consumer choices. This is the case with Colombia, which is one of the first countries in the world to enact a ‘junk food law’ to tax ultra-processed foods by 10%, with a further planned increase to 20% by 2025 [20].

## POLITICAL AND ECONOMIC CONTEXT IN ECUADOR AND THE PROMULGATION OF *DECRETO 645*

Ecuador has moved from a decade of democratic socialism (2007–17), experiencing the second oil boom in its history (gross domestic product grew from USD 51 billion in 2007 to USD 94.47 billion in 2013), to right-wing neoliberalism during the terms of Lenin Moreno (2017–21) and Guillermo Lasso (2021–23) [21]. The former two presidential terms were marked by the COVID-19 pandemic, political instability, a security crisis due to drug trafficking, a shrinking economy, and a shift toward market-oriented policies [22–24]. During Guillermo Lasso’s administration, the Organic Law for Economic Development and Fiscal Sustainability allowed the executive branch to reduce the rates of certain taxes ‘at any time by executive decree’ [25,26]. Thus, on 10 January 2023, Ecuador passed an unprecedented presidential decree called *Decreto 645*, which reduced excise taxes on products harmful to health and the environment, including cigarettes and e-cigarettes, alcoholic beverages, sugary industrial drinks, plastic covers, firearms, and ammunition (**Table 1**). The rationale behind this government decree was to strengthen citizen security, fight against smuggling and informality, and contain the impact of inflation on the economy [27]. However, empirical evidence shows that health taxes have no impact on smuggling, inflation, or the illicit trade of taxed products [25].

**Table 1:** Tax reduction of harmful products based on *Decreto 645* in Ecuador

Product	Tax before *Decreto 645*	Tax with *Decreto 645*	% change reduction
Cigarette	USD 0.17 per unit	USD 0.16 per unit	6
*e-cigarette products*	150%	50%	67
Alcoholic beverages	USD 10.36 per litre of pure alcohol	USD 10.00 per litre of pure alcohol	3.5
SSBs*	USD 0.19 per 100 g of added sugar	USD 0.18 per 100 g of added sugar	5.3
Plastic covers	USD 0.10 per plastic cover	USD 0.08 per plastic cover	20
Firearms and ammunition	300%	30%	90

SSBs – sugar-sweetened beverages

*Corresponds to sugary industrial drinks with more than 25 g of sugar per litre.

Right after *Decreto 645* was released, a group of local health professionals, human rights defenders, communicators, lawyers, retirees, and academics warned about the negative consequences of this decree. Virtual spaces for debate and reflection were convened, and this civil society group called on themselves to remain in resistance as a mechanism to oppose state power to defend the health and well-being of Ecuadorians. This group, called *Colectivo Todos Por la Vida *(translated as ‘Collective All for Life’), was divided into three fronts: a legal team, a communications team dedicated to the dissemination of information, and a group of academics responsible for presenting scientific evidence in a clear, honest, and accessible manner to both judicial powers and the community. As citizens, we had hoped that the local Ministry of Health would initiate a fight to repeal this harmful decree and defend the health of Ecuadorians, but sadly, this was not the case. Thus, *Colectivo Todos Por la Vida *started a crusade in court to defend the health and well-being of the Ecuadorian population. In April 2024, one year after intense constitutional litigation, one of the specialised courts of justice located in Ecuador’s capital, Quito, accepted an appeal in second instance (#17U05202300018), leaving *Decreto 645* without effect. Specifically, this appeal confirmed that *Decreto 645* violated constitutional rights and international regulations ratified by Ecuador for tobacco restriction, healthy nutrition, and human security [26]. This ruling also affirmed the preventive function of health taxes in preventing disease and stopping health systems from being overburdened by adiposity-related cardiovascular diseases, cancers, and diabetes mellitus [28]. Despite this triumph, the same economic groups linked to the tobacco/firearms industry, which vigorously opposed the appeal against *Decreto 645* before the Legal Secretariat of the Presidency, the State Attorney's Office, and the judicial courts, continue advocating to overrule this legal determination and to protect their business interests but this time before the Constitutional Court of Ecuador (CCE). In July 2024, *Colectivo Todos Por la Vida *filed an action of unconstitutionality before the CCE against the Law for Economic Development and Fiscal Sustainability that allowed the President to reduce health taxes [25]. Unfortunately, recent rulings by the CCE – such as admitting extraordinary actions by industries when they are not part of the original judicial process, and rejecting two claims made by *Colectivo Todos Por la Vida*, one on the unconstitutionality of the Organic Law for Economic Development and Fiscal Sustainability that supported *Decreto 645*, and another against the judges who denied the unconstitutionality claim – show that the Court is clearly acting in the commercial interests of these industries and against the well-being of the Ecuadorian people.

## LESSONS FROM *DECRETO 645* FOR POLICYMAKERS AND CIVIL SOCIETY

Five lessons can be learned from this process:

− When they include provisions that roll back human rights, presidential decrees can transform into public policies that negatively impact a country’s population.
− It is possible for social organisations and citizen activists to successfully confront large economic/political interests and powers that threaten life, health, security, and the environment.
− Having a multidisciplinary defence that combines legal strategies, communication, academic evidence, and social mobilisation strengthens the defence capacity in court.
− Latin American and international solidarity is key to strengthening demand capacities and the protection of rights.
− Cooperation between countries and international organisations is essential to face common challenges and guarantee the health and well-being of populations.

As a long-term effect of *Decreto 645* in the Ecuadorian society, local health organisations and civil society have learned how to resist and address commercial determinants of health. This social mobilisation carried out by *Colectivo Todos Por la Vida *was based on three strategies: building empirical evidence to draw attention to the causal magnitude of the health harm caused by *Decreto 645*; employing an ethical argumentation strategy showing that *Decreto 645* is a regressive measure of human rights and against the Ecuadorian Constitution; and on using strategic litigation in court. This case illustrates the complex relationships between executive authority, constitutional rights, public health policy, and civil society advocacy in contemporary Ecuador. While the immediate outcome represents a victory for public health advocates, it also highlights the need for robust institutional safeguards when granting executive powers that could affect public health outcomes.

## CONCLUSIONS

The successful challenge to *Decreto 645* by Ecuador's *Colectivo Todos Por la Vida *represents more than a single victory – it exposes both the fragility and the potential of democratic institutions in Latin America. While civil society successfully confronted executive overreach and corporate interests in this case, the struggle raised fundamental questions about power dynamics in the region. How sustainable is a system in which citizens must constantly mobilise to defend basic public health protections? What does it say about our democratic institutions when corporate interests can so easily influence executive decisions that affect population health? The precedent set by this case demonstrates that organised citizen resistance can effectively challenge powerful interests, but it also underscores a troubling reality: the defence of public health should not depend solely on citizen vigilance. As corporate influence on public policy continues to grow across Latin America, the Ecuadorian experience suggests that we must rethink and restructure the relationship between executive power, corporate interests, and public health governance. The battle over *Decreto 645* may be won, but the broader struggle for institutional reform and genuine public health protection will require sustained attention and systemic change.
